# Cyanobacteria and Eukaryotic Microalgae as Emerging Sources of Antibacterial Peptides

**DOI:** 10.3390/molecules25245804

**Published:** 2020-12-09

**Authors:** Verónica Rojas, Luis Rivas, Constanza Cárdenas, Fanny Guzmán

**Affiliations:** 1 Instituto de Biología, Pontificia Universidad Católica de Valparaíso, Valparaíso 2373223, Chile; 2 Centro de Investigaciones Biológicas Margarita Salas (C.S.I.C), c/Ramiro de Maeztu 9, 28040 Madrid, Spain; luis.rivas@cib.csic.es; 3 Nucleo Biotecnología Curauma, Pontificia Universidad Católica de Valparaíso, Valparaíso 2373223, Chile; constanza.cardenas@pucv.cl (C.C.); fanny.guzman@pucv.cl (F.G.)

**Keywords:** cyanobacteria, microalgae, peptide, antimicrobial, antibacterial activity, bioactive compounds

## Abstract

Cyanobacteria and microalgae are oxygen-producing photosynthetic unicellular organisms encompassing a great diversity of species, which are able to grow under all types of extreme environments and exposed to a wide variety of predators and microbial pathogens. The antibacterial compounds described for these organisms include alkaloids, fatty acids, indoles, macrolides, peptides, phenols, pigments and terpenes, among others. This review presents an overview of antibacterial peptides isolated from cyanobacteria and microalgae, as well as their synergism and mechanisms of action described so far. Antibacterial cyanopeptides belong to different orders, but mainly from Oscillatoriales and Nostocales. Cyanopeptides have different structures but are mainly cyclic peptides. This vast peptide repertoire includes ribosomal and abundant non-ribosomal peptides, evaluated by standard conventional methodologies against pathogenic Gram-negative and Gram-positive bacteria. The antibacterial activity described for microalgal peptides is considerably scarcer, and limited to protein hydrolysates from two *Chlorella* species, and few peptides from *Tetraselmis suecica.* Despite the promising applications of antibacterial peptides and the importance of searching for new natural sources of antibiotics, limitations still persist for their pharmaceutical applications.

## 1. Introduction

The advent of the antimicrobial era are, together with sanitation and increasing access to safe drinking water, among the greatest milestones in public health [1]. Antibiotic therapy affords the control of many infectious diseases, otherwise highly lethal. In addition, it pushes forward the boundaries of many other medical treatments, such as immunosuppressive treatments or successful surgical procedures.

The notion of total elimination of infectious diseases by antibiotic therapies soon turned out to be utopic. Today, the world is facing a deep global antimicrobial resistance (AMR) crisis, with an alarming decrease of effectiveness in antibiotic treatments due to the rising resistance acquired by pathogens [2,3]. The overuse, and often misuse, of antimicrobials in clinics [3], is one of the main reasons of AMR, but not the only one. The induction of antibiotic resistance outside nosocomial settings, strongly associated to the antibiotic use in livestock farming [4], aquaculture [5], and the uncontrolled dumping of antibiotics into the environment [6,7], account for the horizontal transmission of antibiotic-resistance traits out of the nosocomial setting. Furthermore, zoonosis may act as a reservoir for resistant organisms. Altogether, the term “One Health” was coined as a common umbrella to encompass all the resistomes, regardless of their biological source, as responsible for induction of resistance [8,9].

This serious situation is worsened by the deficient pipeline for the development of new antibiotic leads, due to the poor return of investments obtained [10,11]. The magnitude of AMR was recognized by the United Nations General Assembly in 2016, that fostered promising initiatives, as the AMR action fund, a multipartner consortium led by the World Health Organization (WHO) expected to put on the market 2–4 new antibiotics by 2020 (https://www.amractionfund.com/). Yet, the end of this crisis will not be achieved in a short time range [12], so immediate solutions must resort to drug repurposing [13,14], or combination therapies, with a simultaneous multitarget attack to the pathogen [15,16]. Thus, the development of new approaches for anti-infectious diseases, such as bacteriophages, enzybiotics, the focus on virulence factors as targets, or the potentiality of CRiSPR-Cas13, is mandatory and urgent [17,18,19,20,21], not only because of the current and alarming situation, but also because of the feasibility to fight emerging ongoing threats, as the COVID-19 pandemics, concerning bacterial co-infections [22].

Among these forefront candidates on trial, are the antimicrobial peptides (AMPs) (for recent reviews, see [23,24,25,26,27,28,29]), which are ancient chemical weapons in the biological welfare. In unicellular organisms, AMPs help the producer cells to strive against competitors sharing the same ecological niche. In pluricellular organisms, they play a defensive role against invading pathogens. The success of AMPs is endorsed by their ubiquitous presence throughout evolution, crossing taxonomical kingdoms [25], even in those organism endowed with a robust and sophisticated antigen-specific immunity. In pluricellular organisms, AMPs may play additional roles out of their primeval function as deterrent for infection, such as messengers for communication among immune cells, angiogenesis, wound healing, autoimmunity [30,31], their dual role in inflammation [32,33], or even in sleep, among others [34,35,36,37,38].

Until few years ago, the pharmaceutical industry was scarcely receptive to peptide-mediated therapies, mostly due to the high cost for production and their poor ADME (absorption, distribution, metabolism, and excretion) profile, despite their huge potential to cover an extremely broad chemical space, and their structural and functional tuning. This concern was driven by the peptide liability to degradation by proteinases and peptidases present in biological fluids, their sequestration by the cellular matrix and serum components, problematic transport across the membranes, as well as the difficulty of the exogenously administered AMPs to reach an effective concentration at deep tissue or organ locations. Most of these shortcomings were addressed and properly solved in recent years, leading to an increasing number of peptide drugs approved by the Food and Drug Administration (FDA) [39,40,41,42].

This turn of the tide underlies new strategies to overcome the limitations described above, converting peptides into valuable drug candidates: firstly, the decrease in cost by implementation of more efficient and cheaper strategies of synthesis [43,44,45,46,47] or, alternatively, the development of improved production of recombinant peptides [48,49,50,51]; secondly, the improvement of peptide bioavailability by engineering strategies aimed to prevent proteolytic degradation, either by manipulation of their primary sequence by incorporation of unnatural amino acids [52,53], β and γ amino acid peptides [54,55], enantiomeric peptides [56,57] and peptidomimetics [58,59,60,61], or by acquisition of a more stable conformation that secludes or shields the recognition of the cleavage sequence by peptidases (cyclation [46,62,63] and stapled peptides [64]).

In addition, the implementation of nanotechnological vehiculation of the peptides improve their bioavailability by targeting the peptide at the right anatomical or cellular location, preventing peptide waste and off-target effects, as well as avoiding the proteolytic degradation of the peptide. In addition, vehicle degradation may sustain or control a gradual delivery of the peptide at the right site [65,66,67,68]. A greater decrease in the number of peptides entering the pipeline for peptide development is achieved by in silico selection of new and improved prediction tools for candidate selection based on an expected higher effectiveness or decreased toxicity [69,70,71,72,73].

Yet, despite the increase of eukaryotic AMPs that entered into the pipeline and reached different phases in clinical trials [26,27,74], none of them are currently implemented as an over-the-counter drug in the market. In fact, all the AMPs in clinical use are from bacterial origin [75,76,77]: colistin, gramicidin, bacitracin, tyrocidine, the two glycopeptides vancomycin and teicoplanin, the lipopeptide daptomycin A), and the lantibiotic nisin extensively applied as a food preservative.

The cost of an AMPs-based anti-infectious therapy is still significantly higher than for classical antibiotics. However, this drawback is blurred in the case of multiresistant bacteria, being AMPs the last resort drug. Although resistance against eukaryotic AMPs can be induced, their frequency is much lower than for classical antibiotics, due to the high loss of fitness associated [78,79,80]. Nevertheless, a serious clinical concern is the resistance against polymyxin, a lipopeptide used as a last clinical alternative for Gram-negative infections with increase not only in its frequency, but also in its spreading into other bacteria [81,82]. On the other side of the balance, the awareness of the importance of host immune reprogramming by AMPs is a more permanent asset of its overall antimicrobial activity, and presumably, less prone to manipulation by bacterial resistance [27,34].

The search for new natural sources of AMPs has also increased; in this context, microalgae and cyanobacteria have enormous potential as a source of molecules with antimicrobial applications with a high probability of finding new potentially more effective molecules. As a background, these organisms are a source of various chemical substances already characterized, such as peptides, proteins, lipids, vitamins, pigments, carbohydrates, terpenoids, polyunsaturated fatty acids, flavonoids, phenolic compounds, and other organic substances with potential uses as biopharmaceuticals [83].

## 2. Cyanobacteria and Microalgae as Producers of Antibacterial Compounds

These microorganisms are known to be able to survive under all kinds of environmental conditions, terrestrial, saline water and freshwater, and even under extremely competitive environments; moreover, they are exposed to a wide variety of predators and to microbial pathogens, such as bacteria, viruses, and fungi. Their flexible metabolism underlies both their adaptation to a diversity of growth conditions and habitats and their capacity to respond to different environmental stresses and nutrients sources. This versatility can explain the diversity and the number of chemical compounds that have been isolated from them [84,85].

The phylum Cyanobacteria is constituted by photosynthetic bacteria encompassing 1528 species and 1984 taxa grouped under 389 genera [86]. The cyanobacteria are the major oxygen producers and nitrogen fixers, playing an essential role in oceanic phytoplankton, but also, they colonize a wide variety of habitats. They appear as single cells, pluricellular forms, or as symbiotic partners of other animal and plants [87].

There are numerous review articles about marine, freshwater, and terrestrial cyanobacteria, belonging to different families, as a source of antibacterial molecules. This antibacterial activity has been attributed to compounds that belong to quite diverse chemical classes. Those types that present the highest number of antibacterial molecules correspond to alkaloids, fatty acids, pigments, phenolic compounds, and terpenoids; however, the wide range of compounds also includes molecules of another type, such as aromatic compounds, cyclophanes, indole, macrolides, peptides, paracyclophanes, and polyphenyl ethers, among others. Table 1 summarizes the chemical diversity of cyanobacterial molecules with antibacterial activity, and their respective producer cyanobacteria species. Most of the antibacterial assays have been performed in vitro by standard conventional methodologies as minimal inhibitory concentration (MIC) and/or zone of inhibition, against Gram-positive and Gram-negative bacteria pathogenic to humans or to other organisms [83,88,89,90,91,92,93,94,95].

Microalgae are photosynthetic eukaryotic microorganisms and the main producers of oxygen, that constitute the basic components of the ecosystem’s trophic chains, accounting for approximately 40% of photosynthesis on the planet; moreover, they can efficiently assimilate nutrients in a eutrophic water body. Microalgae are not only interesting for their bioproducts, but also for their application in bioremediation of waste waters containing inorganic elements and high metal loads, in biological sequestration of CO_2_, and in the production of renewable energy as biodiesel [96].

Microalgae include a great diversity and complexity of strains, as the result of adaptation carried out through billions of years. These microorganisms colonize every known habitat, but are predominantly found in fresh and marine water. The number of microalgal species is not clearly established, AlgaeBase [97] encompasses 159,173 species that include terrestrial, marine and freshwater organisms, but also marine macroalgae (seaweeds) [96,98,99,100,101]. The major Phyla/class accounting for commercial microalgae are Chlorophyta, Rhodophyta, Haptophyta, Stramenopiles, and Dinophyta [102].

Metabolites from microalgae are extremely diverse, and some of them have been associated with growth inhibition of pathogenic microorganisms. Pratt et al. [103] were the first to isolate a microalgal antibacterial compound from the genus *Chlorella*; this compound, named chlorellin, is a mixture of fatty acids with inhibitory activity on Gram-negative and Gram-positive bacteria. Then, other microalgal antibacterial substances emerged between 1950s and 1980s, such as two chlorophyll a derivatives [89]. Table 2 summarizes the main molecules isolated from microalgae with antibacterial activity. These active chemical compounds include short chain fatty acids, monounsaturated and unsaturated long chain fatty acids, as well as a diversity of other chemical compounds, such as phenols, terpenes, pigments and indoles, acerogenins, alkaloids, macrolides, peptides, and volatile halogenated hydrocarbons [84,89,90,104,105,106,107,108]. Other works reported antibacterial activities in cyanobacterial extracts, mostly with organic solvents, such as those from the diatoms *Skeletonema costatum* and *Chaetoceros pseudocurvisetus* with anti-mycobacterial activity, absent from aqueous extracts, but the responsible metabolites were not identified [109].

Despite the potential of microalgae to produce antibacterial products as novel antibiotics, their development as a natural antibiotic is jeopardized by the small amount of compounds extracted from the producer organisms, the often cumbersome chemical synthesis, the associated toxicity, and in vivo inactivation [110].

## 3. Antibacterial Peptides from Cyanobacteria

Cyanobacteria are an almost endless source of new peptide scaffolds. The peptides are synthesized as secondary metabolites required for a successful strive with other microorganisms, as well as for their astonishing environmental adaptation [118]. The biotechnological and medical potential of cyanobacterial peptides has been frequently reviewed and updated in the literature [88,119,120,121,122,123,124,125].

Since the first description of a cyanobacterial peptide with antibacterial activity, the cyclic peptide schizotrim A isolated from a culture of *Schizothrix* sp. many other peptides have been described. Table 3 summarizes the main cyanobacterial peptides, the producer species, their structure and their effect on known pathogenic target bacteria.

**Figure 1:**
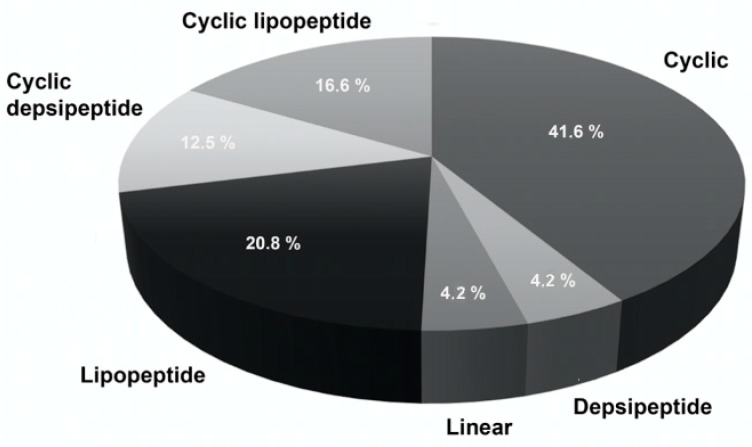
Different types of antibacterial peptides isolated from cyanobacteria.

The antibacterial peptides from cyanobacteria are of different types, although those with a cyclic structure are more frequent (Figure 1). The antibacterial peptides identified peptides belong to different orders of cyanobacteria, being Oscillatoriales and Nostocales the most prolific ones. Inside Oscillatoriales, members of the genus *Lyngbya* are important producers of bioactive peptides with a potential therapeutic use. Four cyclic undecapeptides named lyngbyazothrins A, B, C, and D were identified from the freshwater strain *Lyngbya* sp. The mixtures A/B showed antimicrobial activity only against the Gram-positive bacteria *Micrococcus flavus*, while lyngbyazothrins C/D were active against Gram-negative bacteria (*Escherichia coli*, *Pseudomonas aeruginosa*, and *Serratia marcescens)*, and the Gram-positive *Bacillus subtilis*, although not on methicillin susceptible *Staphylococcus aureus* [92,125,126,127].

Other antibacterial peptides described for the *Lyngbya* genus are the lipopeptide pahayokolide A, and the depsipeptides pitipeptolide A–F. Furthermore, within the Oscillatoriales order, the *Oscillatoria* and the *Phormidium* genus are known producers of antibacterial peptides although with much lower representation than *Lyngbya*. All of them are cyclic peptides. Concerning the Nostocales order, the *Nostoc* genus stands out, with a variety of cyclic and linear antibacterial peptides; *Anabaena* and *Scytonema* genera produced depsipeptides and lipopeptides, respectively [88,94,125,128,129,130,131,132].

The marine cyanobacterium *Prochlorococcus marinus* produces the lantipeptide prochlorosin, with encoding genes distributed throughout its genome. Lantipeptides are a large family of linear and cyclic peptides, ribosomally synthesized as precursor peptides that underwent post-translational modifications, including the formation of lanthionine bridges, heterocyclization, oxidation, methylation, prenylation, and cyclization. The formation of lanthionine bridges in prochlorosin is catalyzed by LanM, a lanthionine synthetase C enzyme [133].

This vast spectrum of peptides produced by cyanobacteria comprises not only the ribosomal synthesis of peptides, including their posttranslational modifications, but also abundant non-ribosomal peptides (NRPs) synthesized by non-ribosomal peptide synthetases (NRPSs) [85,125,134], modular multienzymatic complexes working as an assembly line for amino acid incorporation into the polypeptide chain and, frequently, to their in situ modification. In addition NRPSs may appear associated to polyketide synthases (PKS), forming NRPS-PKS clusters [135,136] broadening even further the variety of chemical motifs incorporated into the polypeptide chain.

NRPSs represent a major class of secondary metabolites in cyanobacteria, with a broad range of biological and pharmacological properties, mostly as antibiotics. It can been speculated that blue-green algae acquired *nrps* genes after the first endosymbiotic process that led to the formation of algae, or algae may subsequent have lost *nrps* genes [101].

The activity on pathogenic bacteria from algae from the orders Chroococcales, Oscillatoriales, Nostocales, and Stigonematales was associated to non-ribosomal pathway involving NRPS, PKS and hybrid NRPS-PKS. Among 50 strains of terrestrial and freshwater cyanobacteria, the species *Cylindrospermopsis raciborskii* 339-T3, *Synechococcus elongatus* PCC7942, *Microcystis aeruginosa* NPCD-1, *Microcystis panniformis* SCP702 and *Fischerella* sp. CENA19 provided the most active extracts, with high activity against *Bacillus subtilis*, and *Salmonella typhimurium* [137].

Microcystins are non-ribosomal cyclic heptapeptides; these toxins are the most commonly found in blooms produced by cyanobacterial genera, such as *Microcystis*, *Anabaena*, *Nodularia*, *Oscillatoria*, *Nostoc*, *Cylindrospermopsis*, *Aphanizomenon*, *Planktothrix*, *Anabaenopsis*, *Synechocystis*, and *Lyngbya*. Aside from them, the strain *Synechocystis aqualitis* M62C produces a microcystin active against *S. aureus*, but devoid of the *mcyB* gene, one of the genes related to the mycrocystin synthesis, whereas the strain *S. aqualitis* M204BG showed microcystin production and *mcyB* gene in its genome, but was inactive against *S. aureus* and *P. aeruginosa* [138].

## 4. Antibacterial Peptides from Microalgae

When compared with the number of cyanobacterial peptides, the number of antibacterial peptides from microalgae is considerably lower. The first report was an antibacterial 30mer peptide purified from the culture of *Stichochrysis immobilis* Pringsheim, active against marine bacteria [145].

The following reports correspond to protein hydrolysates. Sedighi et al. [146], evaluated the antibacterial activity of peptide fractions of the microalga *Chlorella vulgaris*. Protein fraction with 62 kDa were hydrolysates by pepsic digestion and antibacterial activity was determined against *E. coli* CECT 434. The effect of hydrolysate was 8.5 and 1.6 times greater than Chlorella biomass and its proteins, respectively, suggesting that *Chlorella* peptides provoked the cell wall destruction and cell growth inhibition. Furthermore, pepsin hydrolysates and peptide fractions from *Chlorella sorokiniana* displayed antibacterial activity against *E. coli* and *S. aureus* using the agar well diffusion method [147].

Guzmán et al. [148], reported antibacterial peptides from the marine microalgae *Tetraselmis suecica*. The AQ-1766 peptide (LWFYTMWH) obtained from acid extract and the 40% acetonitrile eluted fraction was active against the Gram-negative bacteria *E. coli*, *S. typhimurium*, and *P. aeruginosa*, as well as against the Gram-positive bacterial strains *B. cereus*, methicillin-resistant *S. aureus* (MRSA), *L. monocytogenes* and *M. luteus*. Moreover, the substitutions A4Y (AQ-3000: LWFATMWH), (AQ-3001: LWFYAMWH) and T6M (AQ-3002: LWFYTAWH) increased the antimicrobial activity. Additionally, the lysine analogs: K1L (AQ-3369: KWFYTMWH) and tyrosine Y4K (AQ-3370: LWFKTMWH) exhibited the highest antibacterial activity.

On the other hand, microalgae were also used for transgenic production of alien AMPs, as the bovine AMP lactoferricin by transgenic *Nannochloropsis oculata*. This transfected alga was used as biofunctional food for the medaka fish (*Oryzias latipes)* with an improved survival when challenged with *Vibrio parahaemolyticus* infection [149].

The limited knowledge of antibacterial peptides from microalgae will increase through the use of biotechnological tools such as transcriptomics that will help to understand its genome and its pharmacological interactions with bacteria. Transcriptomic sequencing will provide useful data to identify species with antibiotic potential and pathways for the synthesis of new functional metabolites. The transcriptome of the microalgae *Chrysochromulina tobin* revealed the expression of genes involved in the defense of the algae that encode potential antibiotics, antibiotic extrusion proteins, and novel antibacterial peptides [107].

## 5. Mechanism of Antibacterial Action of Peptides and Compounds of Cyanobacteria and Microalgae

In general, the mechanism of action of cyanobacterial and microalgae peptides against bacterial cells has not yet been established, and further studies are needed to elucidate the biological activity of these antimicrobial peptides [90,147]. For those antibacterial peptides with a clear cationic character, e.g., from microalgae, we may surmise a mechanism of action rather similar to those peptides described in higher eukaryotes; that is, the disruption of the cell membrane after specific insertion into the bacterial cell membrane. Specificity is mostly achieved by the different electrical charge of the external hemilayer of the cell membrane, negative for prokaryotes and lower eukaryotes, zwitterionic in higher eukaryotes. This mechanism has two important consequences; first, the negative electrical charge of the membrane is considered as a pathogen associated molecular pattern, as such, common to many microorganisms, that makes them susceptible to a given peptide. Secondly, as the bactericidal mechanism is based on the stoichiometric interaction of the peptide with the phospholipids of the lipid bilayer, physicochemical characteristics of the peptide, such as charge, size, amphipaticity, and even secondary structure, are more important than the primary sequence of the peptide. For others, their mechanism of action differs from membrane disruption, with involvement of intracellular targets, and specificity achieved by subtle recognition between the peptide and its target.

This is the case for some cyanopeptides, as the cyclic peptides brunsvicamides B and C from *Tychonema* sp., reported as inhibitors of phosphatase B of *Mycobacterium tuberculosis*, or the cyclic depsipeptide scyptolin A, isolated from *Scytonema hofmanni*, an inhibitor of a serine protease working as a transpeptidase involved in the bacterial cell wall biosynthesis for certain pathogenic bacteria [88].

Antibiofilm activity is an appealing asset for an antibacterial candidate, as infections in clinical devices are frequently associated to biofilm formation and higher resistance against antibiotics. The cyclic peptides portoamides produced by *Phormidium* sp. display this activity against marine bacteria such as *Cobetia marina*, *Halomonas aquamarina* and *Pseudoalteromonas atlantica*, by inhibition of ATPase H^+^-transport activity [132]. This activity has a straightforward application as antifouling agents, and their test as antibiofilm compounds for relevant clinical bacteria is pending.

In some cases, structure-activity relationships were obtained with a variable degree of success, either by sequence comparison among similar cyanopeptides from the same or different cyanobacteria, by genetic mutation, or by chemical synthesis. The antibacterial activity of the lipopeptide schizotrin A against *B. subtilis* has been associated to the presence of a proline linked to the 3-amino-2,5,7,8-tetrahydroxy-10-methylundecanoic acid (Aound), and their uptake into the bacterial cell facilitated by the presence of the fatty acid [94,126]. The dipeptide motif formed by a proline residue bound to the amino group of a 2-hydroxy-3 amino-long chain acid residue is shared for other cyanobacterial cyclopeptides, such as scytonemin A from *Scytonema sp* [88,143]. The presence of this fatty acid was also identified in lyngbyazothrins A–D, and this acyl chain at position C-5 is relevant for the antibacterial activity of the peptide.

The amino acid analyses of the cyanopeptides lyngbyazothrins A–D reveal three unusual amino acids identified as 4-methoxyhomophenylalanine in A and C, homophenylalanine in B and D, and 3-amino-2,5,7,8-tetrahydroxy-10-methylundecanoic acid (Aound) in A–D; moreover, C and D have an additional *N*-acetyl-*N*-methyltyrosine unit and it seems that the acyl residue at C-5 plays an important role in antimicrobial activity. Schizotrin A and pahayokolides A and B have sequence similarity to lyngbyazothrins. Schizotrin A presents a 4-methoxyhomophenylalanine (Htm) residue, similar to lyngbyazothrins A and C, bound to the Pro-Aound-Gln-Gly-Pro sequence, common to all of the lyngbyazothrins. The same sequential motif is also found in pahayokolides A and B but, in contrast to schizotrin A, it is linked to an homophenylalanine. Significant differences were found for the remaining five residues of the cyclic systems among the three classes of peptides: Phe-Val-Ser-DeHThr-Ser in schizotrin A, Phe-*Z*-Dhb-Ser-*E*-DhB-Thr in pahayokolides and Pro-allo-Ile-Ser-DeHThr-Thr in lyngbyazothrins. The free hydroxyl group at C-5 of the Aound residue in lyngbyazothrins A and B is substituted by *N*-acetyl-*N*-methyltyrosine in C and D instead of the *N*-butyryl-*N*- methylalanine residue in schizotrin A. On the other hand, schizotrin A and pahayokolide A contain the aliphatic amino acids alanine and leucine, while lyngbyazothrins C and D include the aromatic amino acid tyrosine; it has been proposed that the nature of these amino acids may also account for the activity of lyngbyazothrins against the Gram-negative bacterium *E. coli*, absent in schizotrin and pahayokolide [126].

The ribosomal cyclopeptide aeruginazole A isolated from the cyanobacterium *Microcystis* sp. (IL-323), inhibits the growth of *B. subtilis* and *S. aureus*; in its cyclic structure it contains three subsequent glycine residues plus l-val, l-phe, thiazole-l-val, thiazole-d-leu, d-tyr and thiazole-l-asp. Similarly, aeruginazole DA1497 isolated from *M. aeruginosa*, is a large cyclopeptide with four thiazole (tzl) moieties, having a cyclo-structure of (-(tzl-phe)-gly-ala-ile-(tzl-ala)-ser-(tzl-val)-pro-gly-val-(tzl-leu)-pro-gly-). It seems that the larger size and the greater number of thiazole groups of these compounds may be associated with their bioactivity. Only DA1497 out of the one of five aeruginazole peptides tested (DA1304, DA1274, DA1338 and DA1372) was active against *S. aureus*, even when minor differential sequential variations occurred among the five peptides [92]. The cyclic lipopeptide Trichormamide C from *Oscillatoria* sp. UIC 10,045 is characterized by the presence of three non-proteinogenic α-amino acid residues (*N*-methyl-Ile and two 3-hydroxy-Leu) and one β-amino acid, with a key role on its anti-*M. tuberculosis* activity [125].

The antibacterial mechanisms of microalgal peptides have been scarcely reported to date. The few references on the subject refer to extracts or protein hydrolysates, and not to specific peptides. Microalgal extracts from the species *Leptocylindrus danicus* (FE322) and *L. aporus* (FE332) strongly inhibited the biofilm formation by the bacteria *Staphylococcus epidermidis*, but did not show cytotoxicity by standard antibacterial tests [150]. Tejano et al. [151], reported a higher antibacterial activity on Gram-positive than on Gram-negative bacteria for the pepsin hydrolysate and the peptide fractions from *Chlorella sorokiniana*, likely associated to a hindered penetration of the peptide by the outer membrane.

It has been proposed that microalgal compounds with antibacterial activity are released after the loss of algal integrity, or alternatively induced by the presence of bacteria. For other microalgal compounds involved in a defense mechanism against predators and pathogenic bacteria, it appears that the bacterial cell membrane would be the main site of action. There is some evidence of deleterious effects of fatty acids on the bacterial membrane, causing cell leakage, a reduction in nutrient intake and a reduction in cellular respiration. The antibacterial action of fatty acids can also be mediated by the inhibition of the synthesis of bacterial fatty acids; this effect could be bactericidal or bacteriostatic preventing bacterial multiplication. It has also been reported that antibacterial exometabolites released by *T. suecica* inhibited several *Vibrio* species in vitro causing a rapid decrease in bacterial mobility with cells elongation and vacuolization [84].

Advances in the knowledge of the mechanisms of action underlying the bactericidal activity of peptides from cyanobacteria and microalgae will contribute to the development of these peptides as novel drugs. The role of “omics” techniques in this process, more specially proteomics and peptidomics, will push forward the boundaries for this field [90,147].

## 6. Synergy of Cyanobacterial Peptides

Synergy among AMPs in nature is an important asset in evolution, as it ensures a proper microbicidal function with a spare of components. The issue of microbicidal synergy was extensively addressed for AMPs from higher eukaryotes [152,153,154], as well as for bacteriocins [155]. This synergism may occur under different modalities. A specific molecular recognition between two different AMPs from *Xenopus laevis*, magainin and PGLa, led to the formation of a functional heterodimer as the forming unit of the pore on the bacterial membrane, with an increased lethal permeation over those formed exclusively by a single AMP species [156]. In this case, the two synergic partners shared a single target, but synergy may also arise from two different AMPs each one acting on their own and specific target in a concerted manner. The disruption of bacterial membranes by the human AMP LL-37 allows the histone H2A to gain access into the bacterial cytoplasm to interact with the bacterial genome, and to halt transcription [157]. Synergism is not limited to AMPs as exclusive partners; other actions or compounds may synergize with a given AMP to improve the final microbicidal aftermath. Any AMPs, regardless of its biological origin, able to disrupt the outer membrane of Gram-negative bacteria, which is a strong permeability barrier for small size antibiotics, may likely synergize with them, as described for the bacterial polymyxins B and other AMPS from higher eukaryotes. In addition, small antibiotics may also synergize with AMPs acting mostly through host immunomodulation, as the IDR-1018 peptide [158].

Compared to the synergism described for AMPs, there is a dearth of reports on synergy among cyanobacterial peptides, especially those concerning activity against pathogens of putative clinical interest. Synergism in cyanobacteria was mainly approached from an environmental perspective, where peptides were used by cyanobacteria as allelochemicals for a successful striving for survival among other environmental competitors or plankton grazers.

Portoamides A and B, produced by the mat forming Oscillatoria, act synergistically against the microalgae *C. vulgaris*, *Ankistrodesmus falcatus*, and *Chlamydomonas reinhardtii*, but also against the cyanobacteria *Cylindrospermopsis raciborskii* [159]. *Planktothrix* sp, other cyanobacteria, produced the ribosomal microviridins by ribosomal synthesis, and microcystin-LR, a peptide inhibitor for protein phosphatases 1 and 2A, as well as anabaenopeptins through the non-ribosomal pathway. When cyanobacterial knock-outs for any of these three peptide families were infected with the chytrid parasitic fungus pNIVA-CYA126/8, its virulence was higher on knockouts than on the parental strain [160].

Beyond this environmental frame, synergy among cyanobacterial peptides has also been reported for antifungal activity on species with clinical or phytological importance. Lobocyclamides A and B from *Lyngbya confervoides* act synergistically against *Candida* spp [161], the cyclic undecapeptides laxaphycins A and B from *Anabaena torulosa* against *Aspergillus oryzae* and *Candida albicans* [162], and the same peptides isolated from *Anabaena laxa* against *A. oryzae* [163].

Interestingly, the synergism of cyanobacterial peptides for antitumoral activity has been also reported on human tumoral cell lines for laxaphycins A and B from *A. torulosa*, and for laxaphycins A and B4 from *Hormothamnion enteromorphoides* [162,164]. Protoamides A and B showed synergism on the human cancer cell line H460 [159].

## 7. Other Relevant Functions of Peptides from Cyanobacteria and Microalgae

In addition to their antibacterial activity, the peptides produced by cyanobacteria and microalgae also have other fields of application, being one of the most prominent treatments of cancer. Some representative examples follow:

### 7.1. Antitumoral Activity

The NRPS and NRPS-PKS described on the cyanobacteria of the genus *Nostoc*, were responsible for the production of bioactive peptides (reviewed by Fidor et al. [120]). Among the extensive peptide armamentarium of this genus are cryptophycins; cyclic 16mer depsipeptides are almost exclusively produced by this genus, with activity against different cancer lines. Other peptides of the genus *Nostoc* are the nostocyclopeptides, cyclic heptapeptides characterized by an imino linkage between the first residue (Tyr) and the aldehyde hydrate of the seventh residues. They induce apoptosis in tumoral cell lines, endorsing their feasible use as anticancer compounds.

The heptapeptide LLAPPER (MW = 796.4) was obtained by in vitro gastrointestinal digestion of *P. lutheri* [165]. After its chemical synthesis, the peptide was tested on HT1080 fibrosarcoma cells, with a decrease of the transcript encoding the matrix metalloproteinase B/MMP9, an enzyme involved in the degradation of collagen type IV, through inactivation of the NFκB pathway, as well as blocking of JNK and p38 phosphorylation. Matrix metalloproteinases are involved in the degradation of the extracellular matrix, to promote metastasis in cancer.

Interestingly other cyanobacterial peptides promotes differentiation, as the peptide dubbed PPLF obtained from *P. lutheri* fermentation by Qian et al. [166]. PPLF induced osteoblastic differentiation in the human cell line MG-63 at 50 and 100 µg/mL.

### 7.2. Antihypertensive Activity

Heo et al. [167] hydrolyzed *Spirulina* with a mixture of digestive enzymes (pepsin, trypsin and chymotrypsin). Afterwards, the heptapeptide TMEPGKP (MW = 759) was isolated from a fraction with inhibitory activity on the angiotensin I converting enzyme (ACE). By molecular modelling and functional assays, this peptide resulted as a non-competitive inhibitor of ACE, and decreased the phosphorylation of p38 MAP Kinase (MAPK), of the expression of the inducible nitric oxide synthase (iNOS) with the concomitant decrease of nitric oxide (NO) production. The levels of reactive oxygen species (ROS), and endothelin-1 (ET-1), also decreased after peptide treatment. Altogether, these results make this heptapeptide a good candidate as antihypertensive compound. In addition, the peptide showed an inhibitory effect on the proliferation of EA.hy926 cells at 125 and 250 µM.

Carrizzo et al. [168] described the fractionation of *Spirulina* lysates by a treatment that simulates gastrointestinal digestion (GID). From these extracts, four peptides were obtained and characterized by mass spectrometry (SP(3–6)). In a further step, these peptides were synthesized by Fmoc chemistry and tested for vasorelaxation in an ex vivo model consisting of mouse mesenteric arteria. SP6 peptide (GIVAGDVTPI, MW = 940.52), showed a dose dependent vasorelaxation effect and antihypertensive activity through the activation of endothelial nitric oxide synthase and NO production.

Suetsuna and Chen [169] searched for peptide fractions from the peptic digest of the microalgae *S. platensis* and *C. vulgaris* and to be tested for their antihypertensive activity through ACE inhibition. A total of ten peptides (three to five residues) were isolated, two of them shared by both algae and the other four sequences specific for each species. The two shared tripeptides FAL and AEL showed IC_50_ on ACE of 26.3 and 57.1 µM, respectively. The peptides specific for *S. platensis*: IAE, IAPG, VAF have IC_50_ of 34.7, 11.4 and 35.8 µM, respectively, and the IC_50_ for those exclusively found in *C. vulgaris*: IVVE, AFL, VVPPA, were 315.3, 63.8 and 79.5 µM, respectively.

Hot water extract of the microalgae *Chlorella sorokiniana* was hydrolyzed with proteinase N, and tested for ACE inhibition [170]. Four dipeptides were isolated and sequenced by automated Edman degradation. In a further step, the four peptides were synthesized by solid phase peptide synthesis (SPPS) and tested for their ACE inhibitory activity: WV, VW, IW, and LW with IC_50_ on ACE of 307.6 µM, 0.58 µM, 0.50 µM, and 1.11 µM. Their low IC_50_ values plus their small size make them extremely appealing candidates as hypertensive agents. Furthermore, they were impervious to in vitro gastric digestion.

The peptide YMGLDLK (MW = 839) from the microalgae *Isochrysis galbana* hydrolyzed with alcalase, flavourzyme, pepsin, and trypsin, showed an IC_50_ on ACE of 36.1 µM. Additionally the peptide was stable after incubation with gastric enzymes (pepsin, chymotrypsin and trypsin) [171].

The hydrolysate of the marine microalga, *N. oculata* obtained by digestion with several enzymes, such as pepsin, trypsin, α-chymotrypsin, papain, alcalase, and neutralase were used by Samarakoon et al. [172] in the search for ACE inhibitory peptides. The pepsin hydrolysate exhibited the highest ACE inhibitory activity. From this lysate, two ACE inhibitory peptides were purified: GMNNLTP (MW = 728; IC_50_ = 123 µM) and LEQ (MW = 369; IC_50_ = 173 µM). These peptides were proposed as novel inhibitory agents in the functional food industry.

### 7.3. Anti-Inflammatory Activity

The group of Qian and Jung sought to find active compounds from the marine microalga *Pavlova lutheri* after fermentation with the yeast *Hansenula polymorpha*. They reported the in vitro antioxidant activity of the fermented microalga [173]. In subsequent works [174], reduction of oxidative stress was achieved by the tetrapeptide MGRY (MW = 526), that works as a scavenger for free radicals, with an IC_50_ of 0.285 mM for the DPPH antioxidant test, 0.068 mM for hydroxyl radicals and 0.988 mM for hydrogen peroxide. The peptide also showed inhibitory properties in melanogenesis process when tested in B16F10 melanoma cells. As such, its use was proposed in cosmeceutical and pharmaceutical applications.

### 7.4. Antiviral Activity

Cyanovirin-V, a lectin of 101 residues and two disulfide bridges, showed potent activity against human immunodeficiency viruses (HIV-1 and 2), simian immunodeficiency virus (SIV), and other enveloped viruses [120].

To note, the inhibition of ACE activity described for some cyanopeptides, make them putative candidates for competitive inhibition of the SARS-CoV-2 virus into ACE, the main receptor used for this pandemic virus to infect human cells.

### 7.5. Antifouling Activity

Portoamides are cyclic dodecapeptides from the cyanobacterium *Phormidium* sp. They were used for their antifouling activity with an EC_50_ of 3.16 µM against the settlement of the larvae of the mussel *Mytilus galloprovincialis* [132]. Portoamides were nontoxic against the mussel larvae, therefore a deterrent effect towards surface colonization was instead proposed. The results of previous reports of portoamides’ activity against the microalgae *C. vulgaris* (IC_50_ 12.8 µg/mL), *Ankistrodesmus falcatus* (IC_50_ 24.7 µg/mL) and *C. reinhardtii* (IC_50_ 12.6 µg/mL), and the cyanobacterium *Cylindrospermopsis raciborskii* (IC_50_ 28.4 µg/mL) are also described.

## 8. Synthetic Approach

Synthetic approach has become an almost mandatory companion to the discovery of new peptides. The synthesis of peptides in particular is an area of wide development in the current pharmacology. The main goal of this technique for cyanobacterial and microalgal peptides is to overcome the frequent extremely low amount of isolated natural peptides, that limits further studies on their mechanism of action, on the definition of SAR studies, and of a feasible pharmacological development. Peptides have become valuable drug candidates, largely driven by improvements in their synthetic methodology.

Peptide chemistry has developed on two main areas: biosynthesis and chemical synthesis. A special number of Chemical Reviews dealt with the first strategy, with excellent reviews on the subject and some resulting applications [175,176,177,178].

However, the chemical synthesis is the first-choice approach as it allows to obtain pure products with good yield, needed to establish its activity in an unambiguous way, and pursue the establishment of its mechanism of action.

Solid phase peptide synthesis is a well-established methodology developed more than 50 years ago [179,180]. Nowadays, the Fmoc/tBu strategy is the most used [181,182]. Several approaches have been carried out to optimize the process, to improve its efficiency, and more recently to develop an ecofriendly approach, more compliant with the green chemistry principles [41,42,183,184].

Peptide synthesis can be carried out manually, with the possibility of simultaneously synthesizing many sequences by using the “tea bag” protocol [185,186], or also in an automated way where developments such as microwave [187,188], and ultrasonication [189] procedures shortened the time of synthesis and reduced solvent consumption as well as waste generation, additionally allowing the synthesis of longer peptides.

These methodologies were straightforwardly applied to peptides derived from microalgae, with a lower complexity, and in general devoid of posttranslational modifications, due to their ribosomal origin. In contrast, non-ribosomal peptides from cyanobacteria have a much higher structural and compositional complexity, broadening their chemical space, due to the versatility and diversity provided by non-ribosomal synthesis. Although the synthesis of cyanobacterial peptides requires morecomplex strategies, it has been successfully approached for some peptides.

Inguimbert’s group performed the chemical synthesis of cyclic lipopeptides of the laxaphycin family, isolated from several cyanobacterial species. In a first step, the dodecapeptide laxaphycin B was synthesized [190], and assayed for cytotoxicity against a wide range of cancer cell lines (IC_50_ ranging from 0.2 to 6.0 μM). An automated SPPS with an on-resin “head-to-tail” cyclization of the linear precursor of laxaphycin B (laxaB) was used. Synthesis optimization was achieved by the use of 2-(7-aza-1*H*-benzotriazole-1-yl)-1,1,3,3-tetramethyl uronium hexafluororophosphate (HATU) as a coupling reagent. Finally, the head to tail cyclization was carried out with DIC/Oxyma to prevent epimerization. This strategy was also applied for the synthesis of lyngbyacyclamide A. NMR analysis confirmed that the structures of natural and synthetic peptides were identical. In a later work, the same group synthesized other laxaB analogues [191], by replacement of the non-natural amino acids with commercially available counterparts (2-aminodecanoic acid (Ade): β-alanine, the 3-hydroxyleucines (Hle): threonine, and the 3-hydroxyasparagine (HAsn): asparagine. They also implemented an automated microwave SPPS protocol using the same reagents and conditions determined in the previous work. The cyclization residues were selected according to retrosynthetic analysis, and the final product was purified and characterized as before. Although the peptide obtained did not exactly match the expected results, a synthesis and characterization protocol was established.

The synthesis of the peptide mozamide A, produced by a marine sponge of the genus *Theonella* was approached by Junk and Kazamaier [192]. This peptide is a hydroxylated brunsvicamide, a family of peptides isolated from the cyanobacteria *Tychonema,* but differing in the configuration of Val, Lys and Ile amino acids. Interestingly, the right configuration of these residues was confirmed in the synthetic peptide, and established a synthetic scheme for these types of compounds.

Synthesis of lipocyclopeptides, such as the cyclic undecapeptide trichromamide A (TcA) from the cyanobacteria *Trichormus* sp., was performed by Gaillard et al. [193]. SPPS was carried out on a clorotrytil resin to which the uncommon residue aminodecanoic acid (Ada) was anchored and the cyclization step was made in solution with PyOxym/Oxyma. They reported the synthesis of TcA and a second compound, possibly a diastereomer, with a good yield.

Additionally, multiple approaches can be applied to improve the biological performance of peptides, with a special focus to improve their biological stability. This is achieved, by replacement of amino acids that are not susceptible to enzymatic hydrolysis [194], by structural restriction, by cyclization, or by stapling [46,195].

An interesting research area is related with the use of metallic elements in association with peptides to enhance their activity for applications in biomedicine [196,197,198].

In addition to the development in synthesis, there are also advances in peptide purification and analysis methodologies that include techniques, such as HPLC, MS, circular dichroism (CD), and nuclear magnetic resonance (NMR), which can be helpful in studies of structure-activity relationship or in the determination of mechanisms of action [184,199,200,201].

## 9. Conclusions

The deep global crisis produced by antimicrobial resistance (AMR) has led to the search for new compounds to provide alternative ways for the development of antibacterial agents that exploits mechanisms of action different from traditional antibiotics.

This report has reviewed the wide variety of peptide compounds produced by cyanobacteria and microalgae, both extremely versatile microorganisms, with activity against human and aquatic pathogens, their structural diversity, and their mechanisms of action.

Cyanobacteria produce a wide variety of peptides, due to their extraordinary synthetic plasticity, endowed by their capacity to synthesize not only ribosomal peptides, but also non-ribosomal and polyketide-associated peptides. Additionally, their adaptability makes them easily cultured in the laboratory, requiring a low amount of inorganic nutrients, producing different compounds under different experimental conditions.

In contrast, there is a scarce knowledge on how microalgae face bacterial infection and their antibacterial peptides involved. Despite the wide diversity of microalgal species, only few of them have been cultivated and explored for their biotechnological potential. The insight on their exploitation as an appealing source for novel antimicrobial peptides has just started.

A major advantage for many antimicrobial peptides is their low propensity to generate resistance, due to their multiple mechanisms of action, and the synergy among them. The implementation of cyanobacterial peptides as candidates for antibacterial drugs, required a careful and rational planning where several factors are involved; among these, the selection of the target bacteria, and MIC or MBC expected or required are essential. Concerning the first one, an especial effort is addressed for those species responsible for a higher a health risk against human health, mostly due to the rise of resistance and depletion of alternative antibiotics, according to WHO criteria [202] as those included in the group called ESKAPE [203]. Particular attention should be given for those peptides with a broad spectrum of activity. Concerning activity, an ideal compound should have an IC_50_ 25 μM or 10 μg/mL, whereas for extracts at the initial steps of purification, a standard cut-off should be below 100 μg/mL, similar to the criteria established for natural compounds [204,205,206,207]. In this review, we explored the peptides produced by cyanobacteria and microalgae, mainly as antibacterial, with a special focus on compounds with MIC values below those aforementioned (Table 3), as the most appealing candidates for their further pharmacological development and clinical implementation.

The impressive advances both in isolation and characterization of peptides runs parallel to the development of technologies devoted to these tasks. Among them, new and improved chemical synthesis of peptides, mass spectrometry, “omics” techniques, and bioinformatics tools for in silico selection and identification of feasible starting candidates are crucial. Furthermore, concerning antibacterial assays, the implementation of microfluidics and robotized assays, allow high-throughput screenings with a spare of the peptide required. Likewise, new strategies to improve the stability and bioavailability of peptides were reported, to curb these major shortcuts that jeopardizes the effectiveness of peptides as new drugs. Nowadays, very few peptides derived from cyanobacteria or microalgae overcome the initial steps of the pharmacological development, but it is likely that this is only tip of iceberg for a massive exploitation of these organisms as a new source of antibacterial compounds and a promising alternative for current antibiotics in the future.

## Figures and Tables

**Table 1:** Antibacterial compounds from cyanobacteria.

Cyanobacterial Species	Class of Compound	Reference
	**Alkaloids**	[88,89,92,93,111,112]
*Fischrella* sp.	Eucapsitrione	
*Fischrella ambigua*	Nostocarboline	
*Nostoc* sp.	Tjipanazole A and D	
	12-epi-hapalindole E isonitrile	
*Nostoc spongiaeforme*	Nostocine A	
	**Indole Alkaloids**	
*Fischrella* sp.	Ambiguine A, B, D–I, K and M	
*Fischrella ambigua*	Fischambiguine B	
*Nodularia harveyana*	Northarmane	
*Nostoc insulare*	Norharmane-HCl (9*H*-pyrido(3,4-b) indole-HCl) 4,4-dihydroxybiphenyl	
	**Aromatic Compounds**	[88,92,95,111]
*Fischrella ambigua*	Ambigol A and B	
	**Carbohydrates**	[104]
*Anabaena sphaerica* *Chroococcus turgidus* *Oscillatoria limnetica* *Spirulina platensis*	Polysaccharides	
	**Cyclophanes**	[88,89,93,111]
*Nostoc* sp.	Carbamidocyclophane A–E	
	Nostocyclyne	
*Moorea producens*	**Dicarboximides**	[113]
*(L. majuscula)*	Malyngamide C, I and J	
	**Fatty Acids and Lipids**	[89,90,92,94,108,111]
*Fischerella* sp. *Spirulina platensis* *Phaeodactylum tricornutum* *Oscillatoria redekei* *Scytonema* sp. *Scytoscalarol*	Colioric acid α-dimorphecolic acid γ-linolenic acid	
	**Indanes**	[88]
*Nostoc commune*	4-hydroxy-7-methyl indan-1-one	
	**Lactones**	[111]
*Lyngbya majuscula*	δ-lactone malyngolide	
	**Macrolides**	[88]
*Scytonema* sp.	Scytophycin A and C	
	Tolytoxin	
*Nostoc* sp.	**Paracyclophanes**	[92]
	**Pigments**	[90,91,92,111,114,115]
*Anabaena cylindrica* *Nostoc* sp. *Spirulina platensis* *Salpa fusiformis* *Synechocystis* sp. *Tolypothrix nodosa*	Phycobiliproteins Phycocyanins (PC-B and PC-C) Porphyrins (Tolyporphin)	
	**Phenolic Compounds**	[88,111]
*Anabaena sphaerica* *Chroococcus turgidus* *Oscillatoria limnetica* *Spirulina platensis*	4,4′-hydroxybiphenyl Polyphenols	
	**Polyphenyl Ethers**	[88]
*Leptolyngbya crosbyana*	Crossbyanol A–D	
	**Porphinoids**	[111]
*Tolypothrix nodosa*	Tolyporphin J	
	**Terpenoids**	[88,92,93]
*Nostoc commune*	20-nor-3a-acetoxyabieta-5,7,9,11,13-pentaene	
*Eucapsis* sp.	8-[(5-carboxy-2,9-epoxy) benzyl]-2,5-dihydroxy-1,1,4a,7,8-pentamethyl-1,2,3,4,4a,6,7,8,9,10,10-adodecahydrophenanthrene	
*Microcoleus lacustris*	Abietane	
	Comnostins A–E	
	Norbietane	
	
*Scytonema* sp.	Sesterterpene	
	**Others**	[88]
*Nostoc* sp.	EMTAHDCA 9-ethyliminomethyl-12-(morpholin-4-ylmethoxy)-5,8,13,16–tetraaza–hexacene-2, 3 dicarboxylic acid	

*Fischerella ambigua*	Parsiguine	[92,111,116]

**Table 2:** Antibacterial compounds from microalgae.

Microalgae Species	Class of Compound	Reference
*Dunaliella salina*	**Indolic Derivatives**	[84,89,104]
	β-ionone	
	Neophytadiene	
	**Fatty Acids and Lipids**	
*Chlorella vulgaris* *Chlorella pyrenoidosa* *Chaetoceros muelleri* *Chlorococcum* sp. *Dunaliella salina* *Dunaliella primolecta* *Haematococcus pluvialis* *Navicula delognei* *Phaeodactylum tricornutum* *Planktochlorella nurekis* *Scenedesmus obliquus* *Skeletonema costatum*	Chlorellin Butanoic acid Docosa-pentaenoic acid (DPA) Eicosapentaenoic acid (EPA) Hexadecatrienoic acid (HTA) α-linolenic acid (ALA) Methyl lactic acid Octadecatetraenoic acid Oleic acid Palmitoleic acid Triglycerides	[84,89,90,103,104,105,107,111]
	**Macrolides**	
*Amphidinium* sp.	Amphidinolide Q	[117]
	**Pigments**	
*Isochrysis galbana*	Carotenoids	[84,89,90,104]
	Chlorophyll a derivatives	
	(Pheophytin a and chlorophyllide a)	
	Phycobiliproteins	
	**Terpenoids**	
*Isochrysis galbana (six classes)*	Diterpenoids	[104,106]
	**Others**	
*Phaeocystis* sp.	Acrylic acid	[89]
*Navicula delognei*	Ester	[89]
*Dunaliella salina*	α- and β-ionone	[104]
	Neophytadiene	[84]
	Β-cyclocitral	
	Phytol	
*Haematococcus pluvialis*	Methyl lactate	[104]
*Navicula delognei*	Transphytol ester	[84]
*Haslea ostrearia*	Mareninne	[84]

**Table 3:** Cyanobacterial peptides with antibacterial activity.

Peptide Name	Characteristic	Source	Target Bacteria	Activity ^‡^	Reference
Aeruginazole A	Cyclic	*Microcystis* sp.	*Bacillus subtilis*	MIC = 2.2 µg/mL	[92]
Aeruginazole DA 1497	Cyclic	*Microcystis aeruginosa* TAU	*Staphylococcus aureus*	DIZ 7 mm at 25 µg	[92]
Anachelin H	Depsipeptide	*Anabaena cylindrica* CCAP/2A	*Moxarella catharralis*	MIC = 32 µg/mL	[139]
Antillatoxin B	Lipopeptide	Hawaii and Caribbean collection of cyanobacteria	*Listeria monocytogenes* HPB 2812 and *Staphylococcus aureus* ATCC 29213	MICs = 250 µg/mL	[113]
			*Bacillus cereus* LSPQ 2872	MIC: 130 µg/mL	
Borophycin	Cyclic	*Nostoc linckia* and *N. spongieaforme*,	ND	ND	[88,94]
Brunsvicamides A B and C	Cyclic	*Tychonema* sp.	*Mycobacterium tuberculosis*	IC_50_ = 7.3–8 µM	[88]
Kawaguchipeptin B	Cyclic undecapeptide	*M. aeruginosa* (NIES-88),	*Staphylococcus aureus*	MIC 1 μg/mL	[140]
Laxaphycin A	Lipopeptide	Hawaii and Caribbean collection of cyanobacteria	*Listeria monocytogenes* HPB 2812 and *Bacillus cereus* LSPQ 2872	MIC 250 µg/mL	[113]
			*Staphylococcus aureus* ATCC 29213	MIC = 125 µg/mL	
Laxaphycin B	Lipopeptide	Hawaii and Caribbean collection of cyanobacteria	*Listeria monocytogenes* HPB 2812, *Bacillus cereus* LSPQ 2872 and *Staphylococcus aureus* ATCC 29213	MIC = 250 µg/mL	[113]
Laxaphycin B3	Lipopeptide	Hawaii and Caribbean collection of cyanobacteria	*Bacillus cereus* LSPQ 2872	MIC = 250 µg/mL	[113]
Lyngbyazothrins mixture A/B	Cyclic undecapeptide	*Lyngbya sp* 3691 SAG	*Micrococcus flavus* SBUG 16	DIZ 8 mm at 100 µg (Ref. ampicillin 10 μg, inhibition zone 28 mm)	[92,125,126]
Lyngbyazothrins mixture C/D	Cyclic lipopeptide Cyclic undecapeptide	*Lyngbya* sp. *Lyngbya* sp. 3691 SAG	*Bacillus subtilis* SBUG 14,	DIZ 18 mm at 125 µg (Ref. ampicillin 10 μg, inhibition zone 14 mm)	[92,125,126]
			*Escherichia coli* ATCC 11229	DIZ 18 mm at 100 μg (Ref. ampicillin 50 μg, inhibition zone 26 mm)	
			*E. coli* SBUG 13	DIZ 15 mm at 100 μg (Ref. ampicillin 50 μg, inhibition zone 17 mm)	
			*Pseudomonas aeruginosa* ATCC 27,853	DIZ 8 mm at 100 μg (Ref. gentamycin 25 μg, inhibition zone 26 mm),	
			*Serratia marcescens* SBUG 9	DIZ 8 mm at 200 μg (Ref. ampicillin 10 μg, inhibition zone 28 mm),	
Microcystin	Cyclic heptapeptide	*Synechocystis, Synechococcus* and *Romeria*	*Pseudomonas aeruginosa* ATCC 27,853 and *Staphylococcus aureus* ATCC 25923	DIZ 10.5 ± 0.71 to 14.0 ± 1.41 mm (*)	[94,138]
Muscoride A	Linear	*Nostoc muscorum*	*Bacillus subtilis*	DIZ = 3–6 mm (streptomycin, 7–10 mm; penicillin G, 7–10 mm)	[88,129]
NRPs, PKs and hybrid NRPS-PKS		Brazilian isolates	*Bacillus subtilis* and *Salmonella typhimurium*	34 and 22% of inhibition growth (20 µL/2 mL organic extract)	[137]
Pahayokolide A	Cyclic lipopeptide	*Lyngbya* sp.	*Bacillus megaterium*	MIC = 5.5 µg/mL	[125,131]
			*Bacillus subtilis*	MIC 10 µg/mL	
<3 kDa peptide fraction	Hydrolyzed protein	*Spirulina platensis*	*Escherichia coli*	15.2% at 625 µg/mL	[141]
			*Staphylococcus aureus*	19.6% at 625 µg/mL	
Pitipeptolides A–F	Cyclic depsipeptide	*Lyngbya majuscula*	*Mycobacterium tuberculosis*	DIZ 40 mm at 100 µg/disk	[88,125]
Pitiprolamide	2,2-diMe-3-Hy-hexanoic acid and Dpv-Pro	*L. majuscula*	*Mycobacterium tuberculosis* H37Ra and *Bacillus cereus*	ND	[125,142]
Portoamide	Cyclic	*Phormidium* sp. LEGE 05,292	*Cobetia marina* CECT 4278	23.3% at 6.5 μM	[132]
			*Halomonas aquamarina* CECT 5000	21.0% at 6.5 μM	
			*Pseudoalteromonas atlantica* CECT 570	21.5% at 6.5 μM	
Schyzotrin A	Cyclic lipopeptide	*Schizothrix* sp. TAU strain IL.89-2	*Bacillus subtilis*	DIZ 15 mm at 6.7 nM	[143,144]
Scytonemin A	Lipopeptide	*Scytonema* sp.	*Mycobacterium* sp.	MIC = 1 mg/mL (Ref. gentamycin 0.5 mg/mL)	[128]
Tenuecyclamide A to D	Cyclic hexapeptide	*Nostoc spongieaforme* var tenue	*Bacillus subtilis* Bs1091-1 *Staphylococcus aureus* Sau1091-3 Clinical Laboratory, Ministry of Agriculture, Bet-Dagan, Israel	Disk inhibition zone, values not reported	[88,130]
Tiahuramide C	Cyclic depsipeptide	*L. majuscula*	*Aeromonas salmonicida*	MIC = 6.7 μM	[125]
Trichormamide C	Cyclic lipopeptide	*Oscillatoria sp* UIC 10045	*Mycobacterium tuberculosis*	MIC = 23.8 µg/mL	[125]

**^‡^** MIC: minimal inhibitory concentration; DIZ: diameter inhibition zone (mm); IC_50_: half inhibitory concentration; %: percentage of inhibition. (*) Methanolic extract of *Romeria gracilis* M6C against *Pseudomonas* *aeruginosa*: 10.5 ± 0.71; *Synechocystis aquatilis* M62C against *Staphylococcus* aureus: 11.5 ± 0.71. Ethanolic extract of *R. gracilis* M6C against *P. aeruginosa*: 11.0 ± 1.41; *Synechococcus sp* M94C and M290C against *P. aeruginosa*: 12.5 ± 0.71 and 14.0 ± 1.41 respectively.
